# Characteristics of Polygalacturonate Lyase C from *Bacillus subtilis* 7-3-3 and Its Synergistic Action with PelA in Enzymatic Degumming

**DOI:** 10.1371/journal.pone.0079357

**Published:** 2013-11-13

**Authors:** Mouyong Zou, Xuezhi Li, Jian Zhao, Yinbo Qu

**Affiliations:** State Key Laboratory of Microbial Technology, Shandong University, Jinan, Shandong, P. R. China; RMIT University, Australia

## Abstract

An alkaline polygalacturonate lyase (PGL) from *Bacillus subtilis* 7-3-3, PelC, with diverse depolymerization abilities for different pectin substrates was found. The PGL activity of PelC decreased with increasing degree of methyl esterification of the substrate. PelA and PelC displayed notable synergistic effects in the enzymatic degumming of ramie fibers. Gum loss rates increased by 62% when PelC was used to replace up to three-eighths of the PelA dose (PelC, 60 U g^−1^ ramie fibers). To the best of our knowledge, this study is the first to report the synergistic action of members of polysaccharide lyase families 1 and 3, represented by PelA and PelC, respectively. The present paper provides new insights into the improvement and production of enzymes used in enzymatic degumming.

## Introduction

Polygalacturonate lyases (PGLs, EC 4.2.2.2) catalyze the trans-eliminative cleavage of polygalacturonate, which generates Δ4∶5 unsaturated oligogalacturonates [1]. Three PGL genes, namely, *pelA*, *pelB*, and *pel*C, have been found in *Bacillus subtilis* [2]. Alkaline PGL, mainly referring to PelA, degrades pectins located in the primary cell wall and the middle lamella of higher plants; thus, the enzyme plays an important role in enzymatic degumming [3], [4].

Enzymatic degumming, a potential alternative to chemical degumming, presents several advantages, including flexible operation, limited damage to fibers, easy quality control [5], low energy consumption, and low environmental pollution. However, enzymatic degumming in the textile industry has developed slowly because of the high cost and partial degumming performance of the available enzymes. Most microbial or enzymatic degumming processes are combined with a chemical process to achieve better degumming efficiency and meet the requirements of the textile industry [6], [7]. As such, development of an enzyme system that can enhance the efficiency of the degumming process can promote the industrial-scale applications of enzymatic degumming.

Recent research efforts have focused on the search for proteins that can enhance enzymatic degumming. Some enzyme components, such as xylanase [4], protease [8], and pectinesterase [3], have been reported to enhance PGL degumming strategies. However, few studies have focused on the effect of purified PGL enzyme components on degumming.

*pelC* from *B. subtilis* has been heterologously expressed in *Escherichia coli* and characterized [9]. PelC can depolymerize polygalacturonates and pectins with methyl esterification degrees ranging from 22% to 89% [9]. The three-dimensional (3D) structure of PelC has also been reported [10]. To date, published studies on PelC have focused on its expression and characteristics; few studies have focused on the effects of the enzyme on the degradation and removal of gum from the cell wall of plants. In this study, a *pelC* gene from *B. subtilis* 7-3-3 is heterologously expressed in *Pichia pastoris* GS115. The characteristics and synergistic actions of PelC with PelA on enzyme degumming are also presented.

## Materials and Methods

### Material

Ramie bast fiber was obtained from Yiyang City, Hunan Province, China. The fiber was air-dried and kept in a plastic bag until its use in the experiment.

### Strains and Culture Conditions

*B. subtilis* 7-3-3 was isolated from soil in Shandong University and preserved in the China Center for Type Culture Collection (CCTCC; NO: M20038). *E. coli* DH5α (Beijing Dingguo Biotechnology Co., Ltd., China) was used for plasmid preparation and gene cloning. *E. coli* cells containing plasmids were grown aerobically in Luria-Bertani (LB) medium (10 g L^−1^ peptone, 5 g L^−1^ yeast extract, and 5 g L^−1^ NaCl, pH 6.8–7.0) or on LB agar plates at 37°C and supplemented with 100 µg mL^−1^ ampicillin (Sangon, China).

*P. pastoris* GS115 (Invitrogen, USA) was used to express recombinant PelA and PelC. All *P. pastoris* strains were cultured in a shake flask at 30°C and 200 rpm or on agar plates at 30°C. The media used for the cultivation of *P. pastoris* are as follows: The yeast extract peptone dextrose (YPD) medium contained 20 g L^−1^ peptone, 10 g L^−1^ yeast extract, and 10 g L^−1^ glucose. The minimal dextrose (MD) medium contained 10 g L^−1^ glucose and 13.4 g L^−1^. The yeast nitrogen base (YNB) medium contained 74.6% (NH_4_)_2_SO_4_) and 20 g L^−1^ agar powder. The minimal glycerol medium(MGY), a glycerol complex medium for proliferation, contained 20 g L^−1^ peptone, 10 g L^−1^ yeast extract, 11.8 g L^−1^ KH_2_PO_4_, 3.0 g L^−1^ K_2_HPO_4_·3H_2_O, 13.4 g L^−1^ YNB, 2 mL L^−1^ 500×Biotin, and 10 mL L^−1^ glycerol. The methanol complex medium (MM) used for expression contained the same ingredients as the MGY medium but the glycerol was replaced with 10 mL L^−1^ methanol.

### Assays of PGL Activity and Protein Concentration

PGL activity was determined by measuring the absorbance of unsaturated bonds between C4 and C5 at 235 nm. The reaction mixture contained 2 mL of 0.2% (w/v) polygalacturonic acid (Type P3889, Sigma, USA) in 200 mM glycine–NaOH buffer at pH 9.6 (containing 200 mM glycine, 200 mM NaOH, and 0.44 mM CaCl_2_) and 20 µL of diluted enzyme solution. The reaction mixture was incubated at 45°C for 15 min, and the reaction was terminated by addition of 3 mL of 30 mM phosphoric acid. The product was determined using a spectrophotometer (Shimadzu, UV-2550 PC) at 235 nm. One enzyme unit was defined as the formation of 1 mmol unsaturated polygalacturonic acid per minute, with a molar extinction coefficient of 4600 [11]. Protein concentrations were determined by the Bradford method [12]. The binding of Coomassie Brilliant Blue G-250 to the protein samples caused an absorbance shift and an increase in absorption at 595 nm, which was monitored using a spectrophotometer (Shimadzu, UV-2550 PC).

### Cloning, Modeling of *pelC*, and Phylogenetic Analysis

The *pelC* gene was amplified using primers described in Table 1. The gene was obtained from the *B. subtilis* 7-3-3 genome, cloned to pMD18-T (Takara, Japan), and sequenced in BGI. The *pelC* gene structure was predicted based on a previously published *B. subtilis* PelC (Protein Data Bank code 1ee6) [10] structure. The 3D structures of PelC were modeled using the automated mode of SWISS-MODEL. Homologous proteins of PelC in different species were found using BlastP (http://blast.ncbi.nlm.nih.gov/), and amino acid sequences were aligned using Clustal X [13]. Phylogenetic analysis was performed by the neighbor-joining method using MEGA4 [14], [15].

**Table 1:** Oligonucleotide primers used for the construction of plasmids.

Fragment	Primer	Nucleotide sequence (5′-3′)
**Primers used in cloning of ** ***pelC***		
*pelC*	pelC1	GGCTTGAAAAAAATCGTGTCTATCC
	pelC2	GCGTTAAAATTGAGTGTTGTTGTT
**Primers used in expression of ** ***pelA*** ** and ** ***pelC***		
*pelA*	pelYH1	GGTTACGTAGCTGATTTAGGCCATCAAACGT
	pelYH2	TATGCGGCCGCTTA***ATGATGATGATGATGATG***ATTTAATTTACCAGCACCAGCTT
*pelC*	pelCYH1	GGTTACGTAGCAGACAAAGTGGTGCACGA
	pelCYH2	TATGCGGCCGCTTA***ATGATGATGATGATGATG***AAATTGAGTGTTGTTGTTTTC

Primers were used during PCR to generate various DNA fragments.

### Expression and Purification of PelA and PelC

The coding sequences of *pelA* and *pelC* were amplified using primers described in Table 1. The *pelA* and *pelC* sequences were obtained from the genome of *B. subtilis* 7-3-3 and respectively cloned between the *SnaB*I and *Not*I sites of the pPIC9K expression vector (Invitrogen). Correctly constructed pPIC9K-pelA and pPIC9K-pelC were confirmed by nucleotide sequencing. The recombinant plasmids pPIC9K-pelA and pPIC9K-pelC were linearized by *Mss*I, *Sac*I, and *Sac*I and transformed into the *P. pastoris* GS115 strain for protein expression. Electroporation, selection, and cultivation of recombinant *P. pastoris* were conducted according to a protocol previously reported by Zhuge et al. [16]. The fermentation broths of the transformants were concentrated, loaded onto an His Trap™ FF crude column (GE Healthcare, Sweden), and then eluted with elution buffer containing 300 mM imidazole, 500 mM NaCl, and 20 mM KH_2_PO_4_-K_2_HPO_4_ (pH 7.4) to obtain the PelA and PelC by His-Tag.

### Sodium Dodecyl Sulfate Polyacrylamide Gel Electrophoresis (SDS–PAGE) of Proteins

SDS–PAGE analysis was performed using the supernatants of the cultures or purified protein samples according to a protocol previously reported by Guo et al. [8].

### Deglycosylation of PelC

Purified PelC was diluted and incubated at 37°C for 12 h in a deglycosylation reaction system containing 200 mg of samples, 2 µL 10×G7 buffer (0.5 M sodium phosphate, pH 7.5 at 25°C), 2 µL PNGase F (New England Biolabs, USA), and 6 µL H_2_O. In another method, the PelC was also incubated at 37°C for 12 h in a deglycosylation reaction system containing 300 mg of samples, 2 µL 10×G5 buffer (0.5 mM sodium citrate, pH 5.5 at 25°C), 2 µL Endo H (New England Biolabs), and 6 µL H_2_O.

### Characteristics Assay of PelC

The substrate specificity of PelC was determined by comparing its relative activity using substrates with different methyl esterification degrees (PGA, apple pectin, and 85% pectin with esterification degrees of 0, 70% to75%, and ≥85%, respectively; Sigma, USA). The optimal pH and temperature were determined over pH values ranging from 8.5 to 10.6 and temperatures ranging from 40°C to 75°C, respectively. Thermal stability was determined by incubating the purified enzyme at 30, 40, 50, 60, and 70°C for 8 h in pH 7.0 buffer. The PGL activity of the samples was then determined at 45°C. pH stability was measured through similar steps. The pH gradient was 7.0, 8.0, 9.0, 10.0, and 10.6, and incubation was performed at 4°C.

### Synergistic Action of PelC and PelA on Enzymatic Degumming

The protocol used for enzymatic degumming of ramie fibers was based on descriptions reported in a previous study [11]. The depolymerization capacity of PelA, PelC, and their combinations on different substrates (PGA, apple pectin, and 85% pectin) was also assayed using a 2 mL reaction system (pH 9.6, Gly-NaOH buffer) containing 0.4% (w/v) substrate. The total dose of PGL in each group was 30 mU. Polygalacturonic acid was measured after 15 min at 45°C using a method similar to that employed for the PGL activity assay.

### Observation of Ramie Fibers via Scanning Electron Microscopy (SEM)

SEM observation was used to study changes in the surface of the fibers before and after enzymatic degumming using a JEOL JSM-6700 SEM (JEOL, Japan).

## Results and Discussion

### Phylogenetic Analysis of PelC

**Figure 1:**
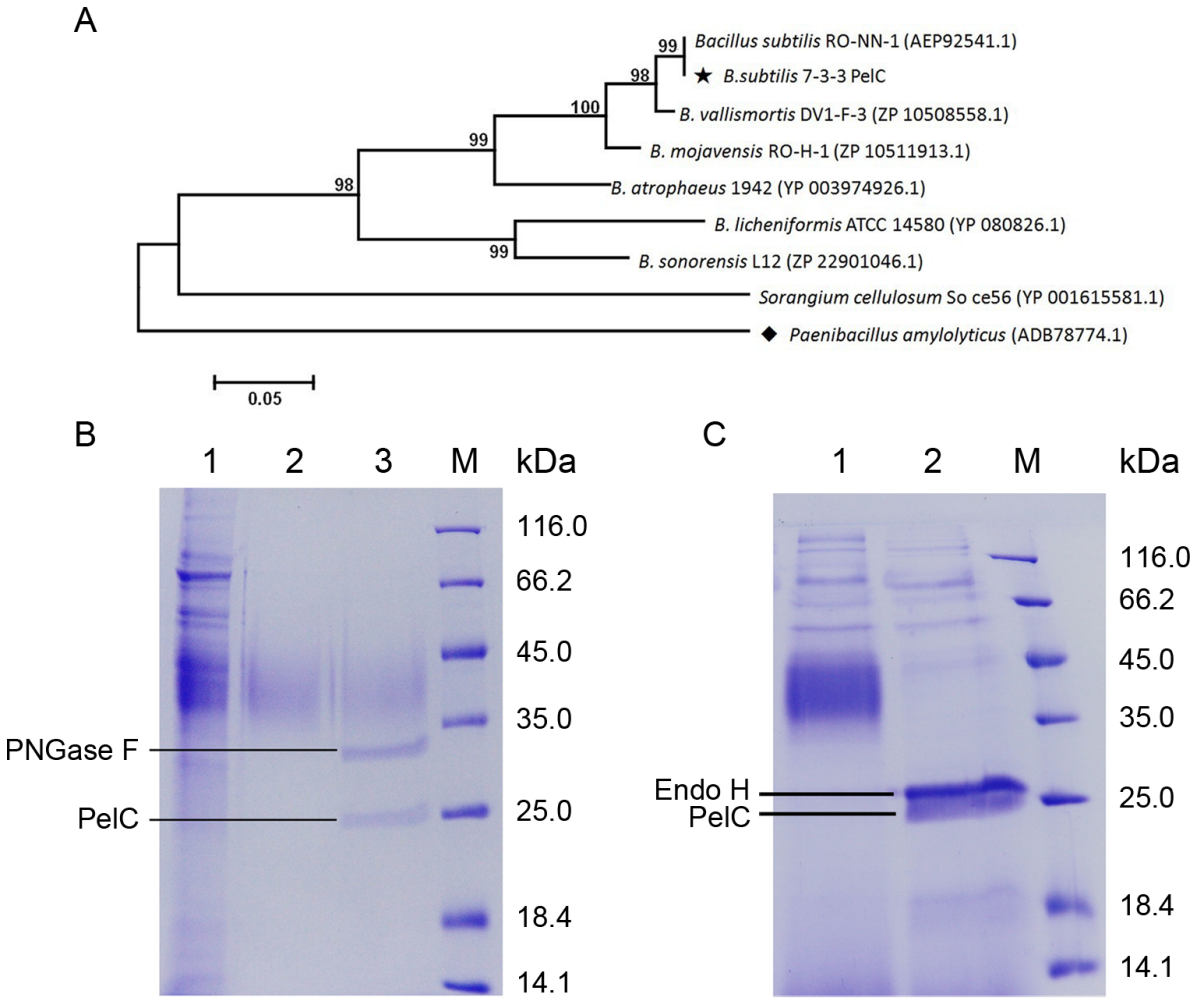
Phylogenetic analysis and SDS-PAGE detection of *B. subtilis* 7-3-3 PelC. (A) Phylogenetic analysis of *B. subtilis* 7-3-3 polygalacturonate lyase PelC (labeled with stars) with its homologous proteins (outgroups labeled with diamonds). (B) SDS-PAGE detection of purified PelC using a His trap™ FF column and deglycosylation by PNGase F. Lane 1, concentrated fermentation broth; Lane 2, purified protein; Lane 3, products of deglycosylation; Lane M, protein molecular weight markers. (C) Deglycosylation of PelC by Endo H. Lane 1, concentrated fermentation broth; Lane 2, products of deglycosylation; Lane M, protein molecular weight markers.

PelC in *B. subtilis* 7-3-3 was classified as a member of polysaccharide lyase (PL) family 3 according to the results of BlastP analysis. Phylogenetic analysis revealed that PelC displays significant homology with pectate lyase in other *Bacillus* species (Fig. 1A). PelC also displayed relatively distant relationships with pectate lyase in *Sorangium cellulosum* and *Paenibacillus amylolyticus* (outgroups).

### Effect of Deglycosylation on PelC

PelC was expressed in *P. pastoris* GS115 and purified from the fermentation broth using a His Trap™ FF column. A trailing band for PelC (Fig. 1B) was observed. The effects of PelC glycosylation on its molecular weight and PGL activity were investigated by deglycosylating the protein by PNGase F. A 25 kDa band approximating the theoretical molecular weight of PelC was observed after deglycosylation, and the PGL activity of the PelC showed limited change (91.4% relative to the untreated specimen). Endo H (29 kDa) was capable of completely removing the glycosyl group of PelC (Fig. 1C). The sequence identity of PelC was obtained via SWISS-MODEL, and the enzyme was found to share 57.87% identity with its templates (1ee6). Sequencing analysis of PelC protein indicated three common N-glycosylation sites (N-X-S/T). All three sites were found in the PelC molecular surface, which suggests that glycosylation of PelC is a normal phenomenon when the protein is heterogeneously expressed in *P. pastoris* GS115.

### Characteristics of PelC

**Figure 2:**
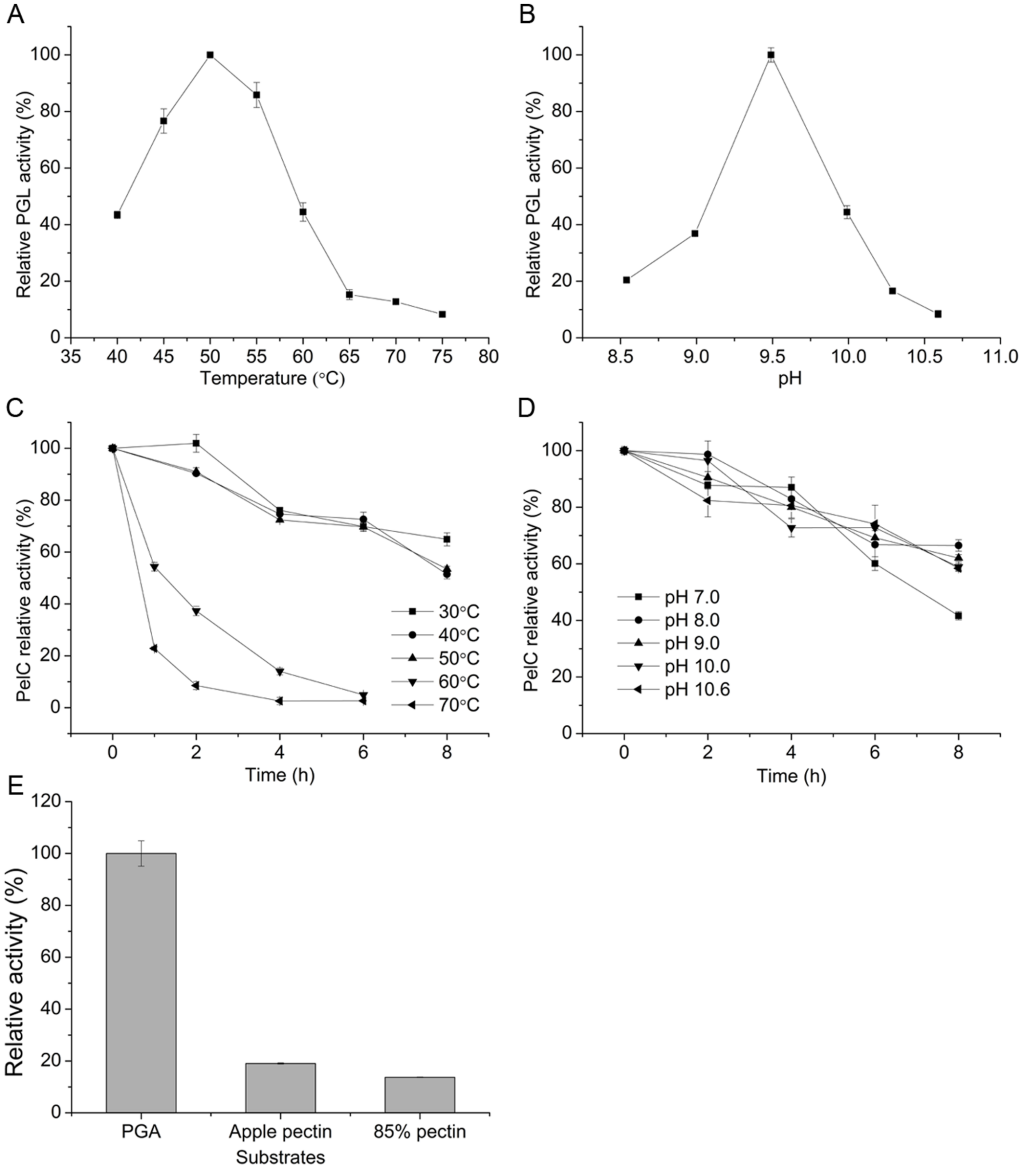
Optimum (A) temperature and (B) pH and (C) thermal and (D) pH stability of PelC. (E) Substrate specificity of PelC.

The optimum temperature and pH of the PelC were determined by measuring the PGL activity at different temperatures and gradient pH buffers. The optimum temperature and pH of PelC were 50°C (Fig. 2A) and 9.5 (Fig. 2B), respectively. PelC showed high activity at temperatures ranging from 45°C to 55°C and remained stable at temperatures below 50°C. Moreover, 64.9% of the enzyme activity was maintained at 30°C for 8 h (Fig. 2C). Fig. 2D shows the pH stability of PelC, in which 41.7%, 62.1%, and 58.4% of the activity of PelC was retained when the protein was incubated at pH 7.0, 9.0, and 10.6, respectively, for 8 h. When measured using the optimal substrate, PGA, the specific activity of PelC was found to be 107.8 U mg^−1^. The PGL activity of PelC decreased with increasing degree of methyl esterification of the substrate (Fig. 2E). Previous reports have shown that PelC can catalyze pectin with degrees of methyl esterification ranging from 22% to 89% and that its optimal substrate is 22% methyl-esterified pectin [9].

### Synergistic Action of PelC with PelA in Enzymatic Degumming

The gum loss rates of ramie fibers obtained in the degumming process using purified PelA or PelC of 160 U g^−1^ were 38.8% and 16.8%, respectively. A mixture of PelA+PelC with a dose ratio of 5∶3 (PelA:PelC) yielded a gum loss rate of 62.9% at a total PGL dosage of 160 U g^−1^ ramie fibers, or an increase of 62%, compared with PelA alone. These results indicate the synergistic action of PelA and PelC in the enzymatic degumming of ramie fibers.

**Figure 3:**
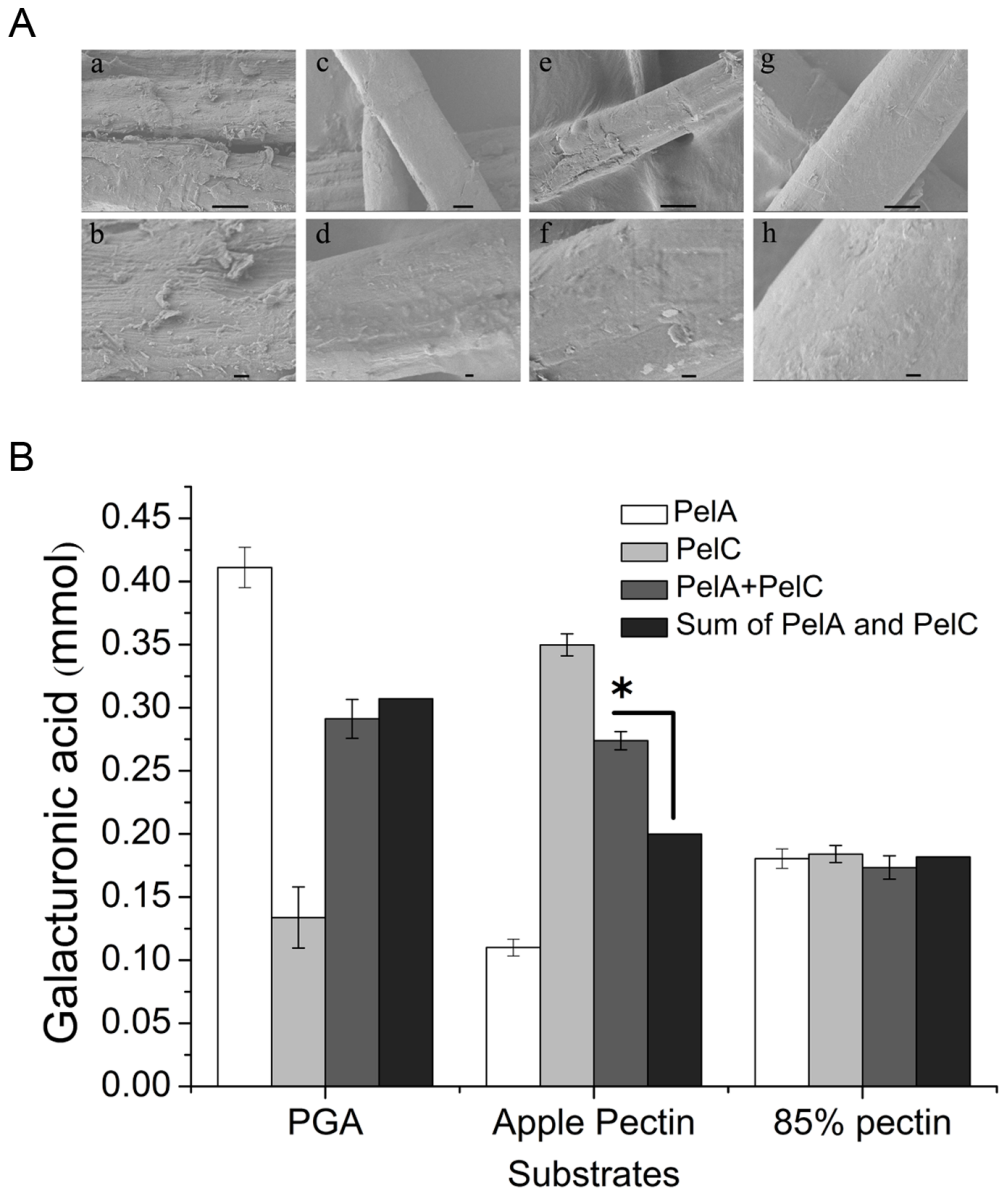
SEM observation of ramie fibers and depolymerization capacity analysis of PelA, PelC, and PelA+PelC. (A) SEM observation of ramie fibers before (a, b) and after treatment with purified PelA (c, d), PelC (e, f), PelA+pelC (g, h). Scale bars: 10 µm (a, c, e, g) and 1 µm (b, d, f, h). (B) Depolymerization capacity of PelA, PelC, and PelA+PelC for different substrates. Enzyme dosage of 30 mU PGL for each group; PelA+PelC, treatment with a mixture of PelA and PelC at PelA:PelC = 5∶3; Sum of PelA and PelC, calculated sum value of PelA and PelC based on its proportion and depolymerization.

SEM observation illustrated that ramie fibers treated with “PelA+PelC” have flatter and smoother surfaces compared with fibers treated by PelA or PelC alone (Fig. 3A).

Different pectin substrates were depolymerized by PelA, PelC, and “PelA+PelC” to further interpret the results. Fig. 3B shows that PelC has higher depolymerization capacity for apple pectin than PelA. The production of oligogalacturonates (degradation product of pectins) in the reaction system of “PelA+PelC” was higher than the sum of production of oligogalacturonates of the PelA- and PelC-only systems (a calculated sum based on the proportion of enzyme doses) when apple pectin is used as a substrate. A statistically significant difference (P<0.001) was detected between “PelA+PelC” and “sum of PelA and PelC” for depolymerization of apple pectin using one-way ANOVA. This result suggests the presence of notable synergistic actions between PelA and PelC in apple pectin depolymerization. However, no synergistic action between PelA and PelC was observed during depolymerization of 85% pectin and PGA, which suggests that “PelA+PelC” has various depolymerization capacities and synergistic actions in different substrates.

To the best of our knowledge, our study is the first to present the diversity of degumming capacities of different members of the PL family, as well as the synergistic action of the purified proteins. Our study helps improve the understanding of the degumming mechanism of PGLs from *B. subtilis* and enhances gum loss rates during multi-enzymatic degumming. Further work is necessary to determine the optimal ratio of PelA and PelC for enzymatic degumming of ramie fibers, and construct an engineering strain with rational expression levels of PelA and PelC to increase the efficiency of enzymatic degumming.

## Conclusion

A polygalacturonate lyase, PelC, was expressed in *P. pastoris* and characterized. PelC exhibited diverse depolymerization abilities for different pectin substrates, and its PGL activity decreased with increasing degree of methyl esterification of the substrate. The diversity of the degumming capacities of PelA and PelC as well as the synergistic effects of the proteins on the enzymatic degumming of ramie fibers were proven. PelC enzymes can be used to design highly effective multi-enzyme mixtures that can help enhance enzymatic degumming.
